# Genome-wide identification and expression analysis of the PEBP gene family in *Ziziphus jujuba* var. *spinosa*

**DOI:** 10.3389/fpls.2025.1700555

**Published:** 2026-01-05

**Authors:** Xiaojun Ma, Xiaozhou Yang, Xinhong Wang, Xiaohan Tang, Xuexiang Li, Dali Geng, Yuqing Ma, Menglin Pu, Jing Shu

**Affiliations:** 1 College of Forestry Engineering, Shandong Agriculture and Engineering University, Jinan, China; 2 College of Agricultural Science and Technology, Shandong Agriculture and Engineering University, Jinan, China

**Keywords:** expression profiling, flowering time, phylogenetic analysis, *Ziziphus jujuba* var. *spinosa*, ZjPEBP

## Abstract

Phosphatidylethanolamine-binding proteins (PEBPs) are known to regulate flowering time and morphogenesis in plants. However, their identification and functions in *Ziziphus jujuba* var. *spinosa* remain uncharacterized. In this study, seven *ZjPEBP* genes were identified and were unevenly distributed across six chromosomes. Phylogenetic analysis classified them into four subfamilies: *FT*-like, *TFL1*-like, *MFT*-like, and *SMFT*-like. The *SMFT*-like subfamily likely originated from horizontal gene transfer (HGT) of prokaryotic origin, exhibiting high sequence similarity to bacteria. In contrast, the remaining six members expanded through dispersed duplication events and possess conserved structures. Cis-acting element analysis suggests that *ZjPEBP* genes may be involved in growth, development, light responsiveness, hormone signaling, and stress adaptation. Reverse transcription quantitative PCR (RT-qPCR) revealed tissue-specific expression patterns among *ZjPEBP* genes. The key flowering regulators *ZjFT* and *ZjTFL1* exhibited antagonistic expression dynamics during fruit-bearing shoot (FBS) development: *ZjFT* expression peaked when FBS reached 2–4 mm in length, coinciding with the initial stage of floral bud differentiation, whereas *ZjTFL1* maintained low expression levels throughout all developmental stages. After flowering, a clear spatiotemporal expression gradient was observed, with *ZjFT* expression in basal leaves being significantly higher than in middle and apical leaves. This expression pattern aligned with the basipetal progression of floral organ differentiation. As the basal and middle sections entered the fruit-setting stage, *ZjFT* expression was markedly downregulated. Under abscisic acid (ABA) treatment, all *ZjPEBP* genes were significantly induced, suggesting their potential involvement in both flowering regulation and ABA signaling pathways. Notably, *ZjSMFT* exhibited the most pronounced response, with expression levels upregulated approximately 400-fold at 24 hours post-treatment. This study provides a systematic characterization of the *ZjPEBP* gene family in sour jujube, laying a solid foundation for further elucidating the molecular mechanisms of flowering regulation and its potential applications in molecular breeding.

## 1 Introduction

Floral transition, marking the switch from vegetative to reproductive growth in higher plants, directly influences yield and fitness (Jung and Müller, 2009). In *Arabidopsis thaliana*, the regulatory mechanisms of flowering have been extensively characterized, with six major pathways identified: the photoperiodic, vernalization, ambient temperature, autonomous, age, and gibberellin pathways. Signals from these pathways converge primarily on *FLOWERING LOCUS T* (*FT*), a member of the phosphatidylethanolamine-binding protein (PEBP) family. FT transmits floral inductive signals to downstream floral identity genes (Corbesier et al., 2007; Amasino, 2010).

The PEBP family, harboring a highly conserved PEBP domain, is widely distributed across prokaryotes, archaea, and eukaryotes. In plants, this family regulates key biological processes including flowering time, seed development and dormancy, and plant architecture (Zhu et al., 2021). Based on phylogenetic relationships, plant PEBP genes are classified into three subfamilies: *FT*-like, *TERMINAL FLOWER1* (*TFL1*)-like, and *MOTHER OF FT AND TFL1* (*MFT*)-like (Chardon and Damerval, 2005; Danilevskaya et al., 2008). The *MFT*-like clade is the ancestor of all *PEBP* genes (Hedman et al., 2009). In *A. thaliana*, it primarily regulates seed germination (Xi et al., 2010; Nakamura et al., 2011), while exhibiting weak floral inductive activity (Yoo et al., 2004). *FT*-like and *TFL1*-like genes function in controlling flowering time (Kobayashi et al., 1999).

The *A. thaliana FT*-like subfamily comprises two members, *FT* and its paralog *TWIN SISTER OF FT* (*TSF*), which redundantly promote flowering with *FT* being dominant (Michaels et al., 2005; Mathieu et al., 2007). FT protein functions as a mobile florigen synthesized in leaf phloem companion cells and transported to the shoot apical meristem (SAM), where it promotes inflorescence meristem formation and subsequent floral transition (Corbesier et al., 2007; Mathieu et al., 2007; Tamaki et al., 2007). Furthermore, *FT* homologs have been identified in a wide range of plant species, and their essential role in flowering regulation is well established across diverse taxonomic groups. For instance, *FT* homologs promote floral transition in both annual plants, such as rice (Tamaki et al., 2007) and tomato (Lifschitz et al., 2006), and perennial woody fruit trees, including apple (Kotoda et al., 2010), citrus (Endo et al., 2005), and poplar (Zhang et al., 2010). Beyond this conserved function in flowering, *FT*-like genes are involved in a variety of other developmental processes. For example, in *A. thaliana*, *FT* regulates stomatal opening and modulates lateral branch growth (Hiraoka et al., 2012; Hassidim et al., 2017). Similarly, *Eriobotrya japonica FT1* influences bud germination and leaf development in loquat (Reig et al., 2017); in potato, an *FT* homolog controls tuberization (Abelenda et al., 2016; Jing et al., 2023); and in onion, *AcFT1* and *AcFT4* act antagonistically to regulate bulb formation (Lee et al., 2013). *TFL1*-like subfamily includes *TFL1*、;*BROTHER OF FT AND TFL1* (*BFT*), and *A. thaliana CENTRORADIALIS* (*ATC*), all delaying flowering. *TFL1* is a major regulator maintaining inflorescence meristem indeterminacy (Shannon and Meeks-Wagner, 1991). In SAM, TFL1 delays floral transition by competitively binding to the bZIP transcription factor FD/FDP against FT (Abe et al., 2005; Hanano and Goto, 2011; Zhu et al., 2020). *BFT* acts redundantly with *TFL1* in regulating inflorescence development (Yoo et al., 2010) and participates in stress responses (Chung et al., 2010). *ATC*, a short-day-induced floral inhibitor, moves via the vasculature to the SAM to antagonize FT-mediated flowering (Huang et al., 2012).

Recent studies have revealed a phylogenetically distinct fourth clade within the plant PEBP gene family, which exhibits a distant phylogenetic relationship with the canonical *MFT*−like, *FT*−like, and *TFL1*−like subfamilies (Bellinazzo et al., 2024; Burton et al., 2025). This clade was designated *STEPMOTHER OF FT AND TFL1* (*SMFT*) by Bellinazzo et al. (2024) and *Sibling of FT/TFL* (*SFT*) by Burton et al. (2025). It clusters with YbhB/YbcL proteins from prokaryotes, suggesting a likely origin from an ancient horizontal gene transfer (HGT) event (Bellinazzo et al., 2024). In *A. thaliana*, *SMFT* (AT5G01300) has been demonstrated to function in abiotic stress response and the regulation of seed germination (Bellinazzo et al., 2024). Furthermore, *SMFT* homologs have been identified in various plant species, including cucurbit crops (Fan et al., 2024), *Brassica juncea* var. *Tumida* (He et al., 2022) and *Brassica oleracea* (Sheng et al., 2020).

*Ziziphus jujuba* var. *spinosa* (sour jujube), a member of the Rhamnaceae family, is the wild progenitor of cultivated Chinese jujube. It exhibits exceptional tolerance to saline-alkali soils, drought, and poor nutrition. Its seeds, known as *Suanzaoren* in traditional Chinese medicine, have been used for millennia to treat insomnia. Unlike most perennial fruit trees, sour jujube has an extremely short juvenile phase, flowering within the first year of planting and completing its entire lifecycle within a single growing season (Meir et al., 2016; Meng et al., 2020). Notably, flower bud differentiation occurs in a “continuous differentiation” pattern: flower buds initiate when fruit-bearing shoot (FBS) reach 2–3 mm in length, with differentiation progressing basipetally as FBS elongate until growth cessation (Qu and Wang, 1993). While enabling early fruiting, this trait causes severe overlap between reproductive and vegetative growth, triggering source-sink competition for photoassimilates. Consequently, abnormal floral development, concentrated flower drop, and extremely low fruit-setting rates occur in jujube. Therefore, elucidating the molecular mechanisms by which the *PEBP* gene family regulates the unique flowering behavior in sour jujube is essential for understanding its reproductive development and enhancing yield.

The PEBP gene family, a key regulator of floral transition, has been systematically characterized in model plants like *A. thaliana* (Wickland and Hanzawa, 2015), tomato (Sun et al., 2023), apple (Zhang et al., 2021), pineapple (Zhang et al., 2023), rice (Zhao et al., 2022), and maize (Danilevskaya et al., 2008). However, its genome-wide identification, evolutionary features, and biological functions in sour jujube remain unexplored. Given sour jujube’s environmental resilience and medicinal value, deciphering its flowering mechanisms is vital for its genetic improvement. In this study, we identified and characterized seven *ZjPEBP* genes in sour jujube, which include one *FT*-like, three *TFL1*-like, two *MFT*-like, and one putative *SMFT*-like member. A systematic analysis was conducted to investigate their phylogenetic relationships, chromosomal distribution, gene structures, conserved motifs, synteny, and cis-acting regulatory elements. Furthermore, we performed expression profiling of *ZjPEBP* members across various tissues and under abscisic acid (ABA) treatment using reverse transcription quantitative PCR (RT-qPCR). Given the central role of *FT*/*TFL1* genes in flowering regulation and sour jujube’s unique continuous flowering pattern, we specifically characterized the expression dynamics of *ZjFT* and *ZjTFL1* at different developmental stages of FBS. This work provides a comprehensive genomic and functional overview of the PEBP gene family in sour jujube, establishing a solid foundation for elucidating the molecular mechanisms underlying its regulation of distinctive flowering traits.

## 2 Materials and methods

### 2.1 Identification of PEBP family members in *Z. jujuba* var. *spinosa*

To identify members of the PEBP gene family in sour jujube, the whole-genome annotation data were obtained from figshare (https://figshare.com/s/ad5d747ccc2ccbb2b65b) (Li et al., 2024). The sequences of 7 A*. thaliana* PEBP proteins were obtained from TAIR (https://www.arabidopsis.org/), and sequences from other species were obtained from Phytozome and Genbank. The AtPEBP protein sequences were used as queries to perform BLASTp searches (E-value ≤ 1.0E-5) against the entire sour jujube proteome. Concurrently, the HMMER program was employed to search for homologous proteins in sour jujube using the Hidden Markov Model (HMM) of the PEBP domain (PF01161) from the Pfam database (E-value ≤ 1.0E-5). Candidate sequences from both methods were submitted to the NCBI CDD and SMART databases to verify the presence of the conserved PEBP domain.

To correct the gene models for *ZjPEBP* members with an incomplete PEBP domain annotation, we utilized publicly available transcriptome data. RNA-seq reads for sour jujube (accessions: SRR9089009–SRR9089012) were downloaded from the NCBI SRA database. The SRA files were converted to FASTQ format using the Convert SRA to Fastq Files tool within TBtools (Chen et al., 2023). The reads were then aligned to the reference genome, and sorted BAM files were generated using SAMtools sort. The alignments were visually inspected in IGV-GSAman to manually correct the exon–intron structure of the target gene annotations. All corrections were subsequently validated by PCR amplification. The primer sequences used are listed in **Supplementary Table S1**.

### 2.2 Chromosomal localization and protein physicochemical property analysis

Based on the sour jujube genome annotation, TBtools was used to map the chromosomal locations of *ZjPEBP* genes (Chen et al., 2023). The physicochemical properties of the ZjPEBP proteins, including theoretical isoelectric point (pI) and molecular weight (MW), were predicted using ProtParam (https://web.expasy.org/protparam/) (Duvaud et al., 2021). The secondary structure of ZjPEBP was predicted using SOPMA (https://npsa.lyon.inserm.fr/cgi-bin/npsa_automat.pl?page=/NPSA/npsa_sopma.html). The three-dimensional structure of ZjPEBP was simulated by an online protein structure homology-modeling server SWISS-MODEL (Waterhouse et al., 2018). Subcellular localization of the ZjPEBP proteins was predicted using Plant-mPLoc (http://www.csbio.sjtu.edu.cn/bioinf/plant-multi/) (Chou and Shen, 2008).

### 2.3 Multiple sequence alignment and phylogenetic analysis

Multiple sequence alignment of 154 PEBP protein sequences from 16 species (*Z. jujuba* var. *Spinosa*, *A. thaliana*, *Macadamia integrifolia*, *Malus domestica*, *Prunus persica*, *Solanum lycopersicum*, *Musa acuminata*, *Oryza sativa*, *Zea mays*, *Cycas revoluta*, *Ginkgo biloba*, *Picea abies*, *Podocarpus macrophyllus*, *Chlorobiota bacterium*, *Myxococcota bacterium*, *Paraburkholderia atlantica*) was performed using the MUSCLE method with default parameters in MEGA 11.0 (Tamura et al., 2021). The phylogenetic tree was constructed using the Maximum-Likelihood (ML) method with 1000 bootstrap replicates. The resulting phylogenetic tree was visualized using iTOL (https://itol.embl.de). The alignment results for the sour jujube ZjPEBP protein sequences were visualized using ESPript 3.0 (https://espript.ibcp.fr/ESPript/ESPript/index.php) (Robert and Gouet, 2014).

### 2.4 Gene structure and conserved motif analysis

Based on the sour jujube genome annotation, TBtools was used to generate schematic diagrams illustrating the exon-intron structures of the *ZjPEBP* (Chen et al., 2023). Conserved motifs in the ZjPEBP were predicted using MEME (https://meme-suite.org/meme/tools/meme) with the maximum number of motifs set to five and the motif distribution mode set to Zero or One Occurrence Per Sequence (zoops) (Bailey et al., 2015).

### 2.5 Collinearity analysis

Intra-species and inter-species collinearity analyses of the sour jujube *ZjPEBP* gene family members were conducted using the McScanX plugin within TBtools, with an E-value ≤ 1.0E-10 (Chen et al., 2023).

### 2.6 Cis-acting element analysis

TBtools was used to extract potential promoter sequences (2000 bp upstream of the transcription start site) of the sour jujube *ZjPEBP* genes. Cis-acting regulatory elements within these promoter regions were predicted using the PlantCARE online database (http://bioinformatics.psb.ugent.be/webtools/plantcare/html/) (Lescot et al., 2002). The results were visualized using GraphPad Prism 9.0.0.

### 2.7 Plant materials and treatments

Healthy perennial sour jujube plants were cultivated at the experimental base of Shandong Agriculture and Engineering University. From April 10, 2025 (bud break initiation), newly sprouted FBS were collected daily at 17:00, categorized by length into six developmental stages: 0–2 mm, 2–4 mm, 4–6 mm, 6–10 mm, 10–15 mm, and 15–20 mm. Following flowering on May 14, 2025, root, stem, leaf, and flower tissues were harvested. Additionally, leaves from the apical, middle, and basal sections of FBS were collected at 14-day intervals.

To determine the response of *ZjPEBP* genes to ABA treatment, sour jujube seeds were germinated at 25 °C under a 16 h light/8 h dark photoperiod. Seedlings were transferred to 9 cm pots (vermiculite: nutrient soil = 1:1) and grown until the six-leaf stage. Uniform seedlings were selected, and a 100 μmol/L ABA solution was applied to the soil around the roots. Leaf samples were collected at 0, 6, 12, and 24 h after treatment.

For all samples described above, three biological replicates were established, each replicate consisted of a pooled sample from five individual plants. Collected samples were immediately frozen in liquid nitrogen and stored at -80 °C until RNA extraction.

### 2.8 RNA extraction

Total RNA was extracted from each sample using a Total RNA Extraction Kit (Vazyme, Nanjing, China). RNA concentration was measured using a NanoDrop spectrophotometer (Thermo Fisher Scientific, USA). 1 μg of total RNA was used for genomic DNA removal and first-strand cDNA synthesis using the HiScript III RT SuperMix (+gDNA wiper) (Vazyme, Nanjing, China). cDNA products were stored at -20 °C for subsequent use.

### 2.9 Gene expression analysis

The expression levels of *ZjPEBP* genes were analyzed using RT-qPCR with three biological replicates, each consisting of three technical replicates. The *Actin* gene served as the internal reference. Reactions were performed using ChamQ Universal SYBR qPCR Master Mix (Vazyme, Nanjing, China) on a QuantStudio 5 system (Thermo Fisher Scientific, USA). Relative gene expression levels were calculated using the 2^-ΔΔCt^ method. One-way analysis of variance (ANOVA) followed by Tukey’s HSD multiple comparison test (P < 0.05) was performed using IBM SPSS Statistics 27.0 software. Data visualization was conducted using GraphPad Prism 9.0.0. Primer sequences used are listed in **Supplementary Table S1**.

## 3 Results and analysis

### 3.1 Identification of ZjPEBP family members in *Z. jujuba* var. *spinosa*

Seven candidate *PEBP* genes were identified in the sour jujube genome through BLASTp and HMMER searches (**Supplementary Table S2**). Validation of conserved domain integrity via NCBI CDD and SMART databases (**Supplementary Table S3**), combined with transcriptome data and PCR amplification verification (**Supplementary Figure S1**), confirmed that all seven candidates encode typical PEBP domains. These genes were unevenly distributed across six chromosomes (**Supplementary Figure S2**). Protein physicochemical analysis ZjPEBP proteins range in length from 167 to 176 aa, with molecular weights between 18.32 and 19.63 kDa (**Table 1**). Except for ZjMFT1 and ZjSMFT, all other ZjPEBP proteins exhibited a theoretical pI >7 and are therefore classified as alkaline. The predicted Grand average of hydropathicity (GRAVY) values were negative for all ZjPEBPs, confirming their hydrophilic nature. Secondary structure prediction demonstrated a predominance of random coils in all ZjPEBP proteins (**Supplementary Figure S3A**). Given the functional relevance of three-dimensional structures, we modeled ZjPEBP tertiary structures using SWISS-MODEL (**Supplementary Figure S3B**). These models revealed conserved structural architectures, providing a structural basis for functional similarity. Subcellular localization predictions indicated nuclear localization for ZjFT, ZjBFT and ZjSMFT; cytoplasmic localization for ZjCEN and ZjTFL1; and dual cytoplasmic/nuclear localization for ZjMFT1 and ZjMFT2.

**Table 1:** List of seven ZjPEBP family in *Z. jujuba* var. *spinosa*.

No.	Gene name	Gene ID ^a^	Genomic location	Protein (aa)	Mw (kDa)	pI	GRAVY	Subcellular localization
1	ZjMFT1	ZspiChr1G00013400.1	chr 1	176	19.54	6.3	-0.348	Cytoplasm/Nucleus
2	ZjCEN	ZspiChr2G00164510.1	chr 2	172	19.51	9.37	-0.272	Cytoplasm
3	ZjFT	ZspiChr2G00168660.1	chr 2	174	19.63	7.75	-0.345	Nucleus
4	ZjBFT	ZspiChr3G00241960.1	chr 3	173	19.37	9.34	-0.29	Nucleus
5	ZjMFT2	ZspiChr5G00104630.1	chr 5	172	18.83	7.88	-0.045	Cytoplasm/Nucleus
6	ZjSMFT	ZspiChr11G00202530.1	chr 11	167	18.32	5.03	-0.375	Nucleus
7	ZjTFL1	ZspiChr12G00251830.1	chr 12	172	19.40	8.89	-0.328	Cytoplasm

a The gene ID, in figshare.

### 3.2 Phylogenetic analysis and multiple sequence alignment of ZjPEBP proteins

**Figure 1:**
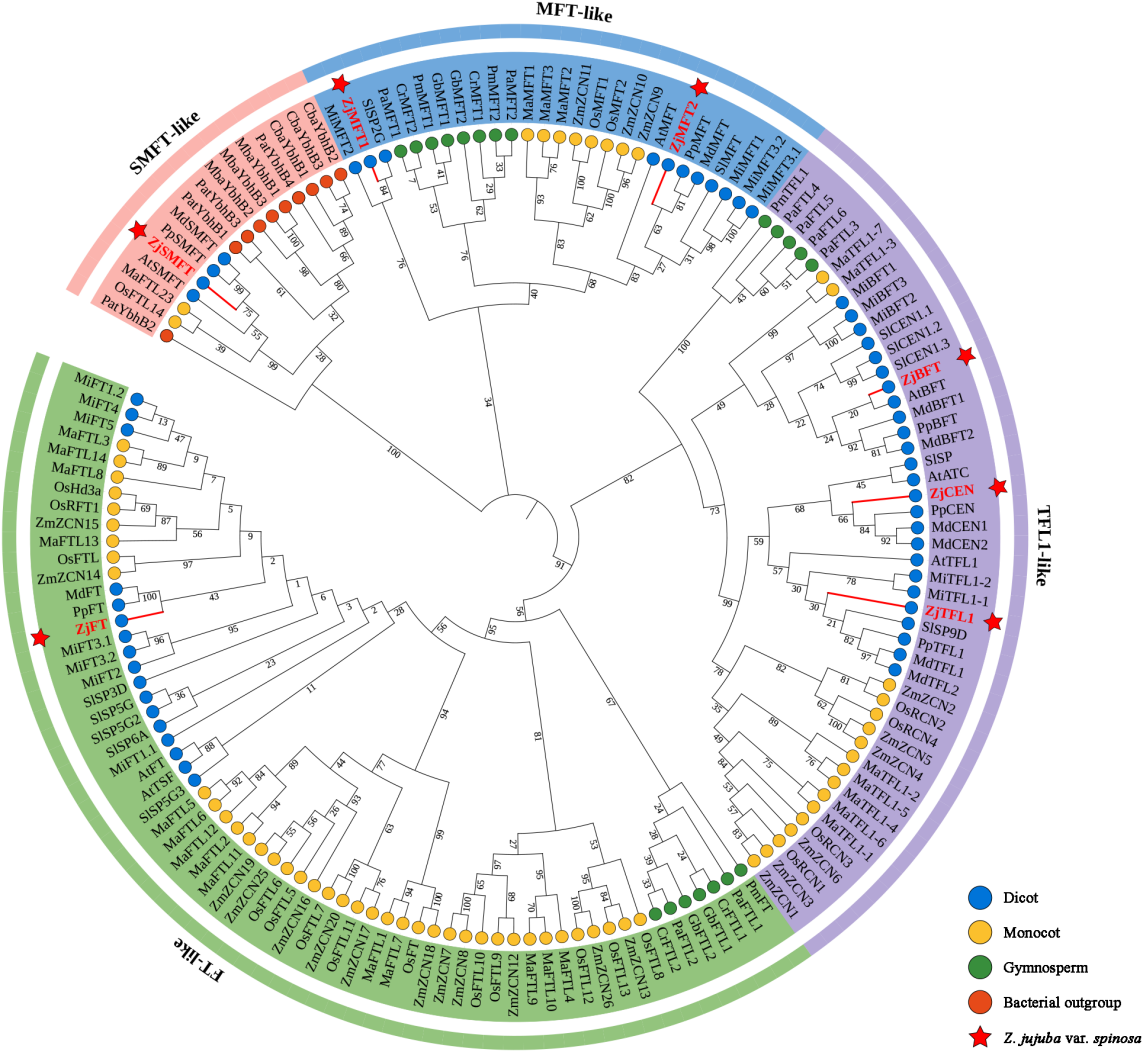
Phylogenetic analysis of PEBP family proteins. ML tree of 154 PEBP proteins from *Z. jujuba* var. *Spinosa*, *A. thaliana*, *M. integrifolia*, *M. domestica*, P. persica, *S. lycopersicum*, *M. acuminata*, *O. sativa*, *Z. mays*, *C. revoluta*, *G. biloba*, *P. abies*, *P. macrophyllus*, *C. bacterium*, *M. bacterium*, and *P. atlantica*. *FT*-like, *TFL1*-like, *MFT*-like, and *SMFT*-like subfamilies are marked with green, purple, blue, and pink background bands, respectively. ZjPEBP members are highlighted in red font with red pentagrams at terminals. Dicots, monocots, gymnosperms, and bacterial outgroups are denoted by blue, yellow, green, and orange circles, respectively.

To elucidate the classification and evolutionary relationships of the sour jujube ZjPEBP family, a phylogenetic tree was constructed using 154 PEBP protein sequences from 16 species with previously characterized PEBP gene families, including five dicot species, three monocot species, four gymnosperm species, and three bacterial species (**Figure 1**, **Supplementary Table S4**). Our phylogenetic analysis revealed that the PEBP family diverged into four distinct clades. These were designated as *FT*-like, *TFL1*-like, *MFT*-like, and *SMFT*-like, a classification consistent with previous reports (Chardon and Damerval, 2005; Bellinazzo et al., 2024). Based on phylogenetic positions and evolutionary relationships, the sour jujube ZjPEBP was systematically named (**Table 1**, **Figure 1**). The sour jujube PEBP family comprises three *TFL1*-like members (ZjCEN, ZjBFT, ZjTFL1), two *MFT*-like members (ZjMFT1, ZjMFT2), one *FT*-like member (ZjFT), and one *SMFT*-like member (ZjSMFT). In most clades, ZjPEBP members clustered closely with those from Rosaceae species (*M. domestica*, *P. persica*), suggesting a general phylogenetic affinity with rosaceous plants.

### 3.3 Analysis of conserved motifs, domains, and gene structures in ZjPEBP family

**Figure 2:**
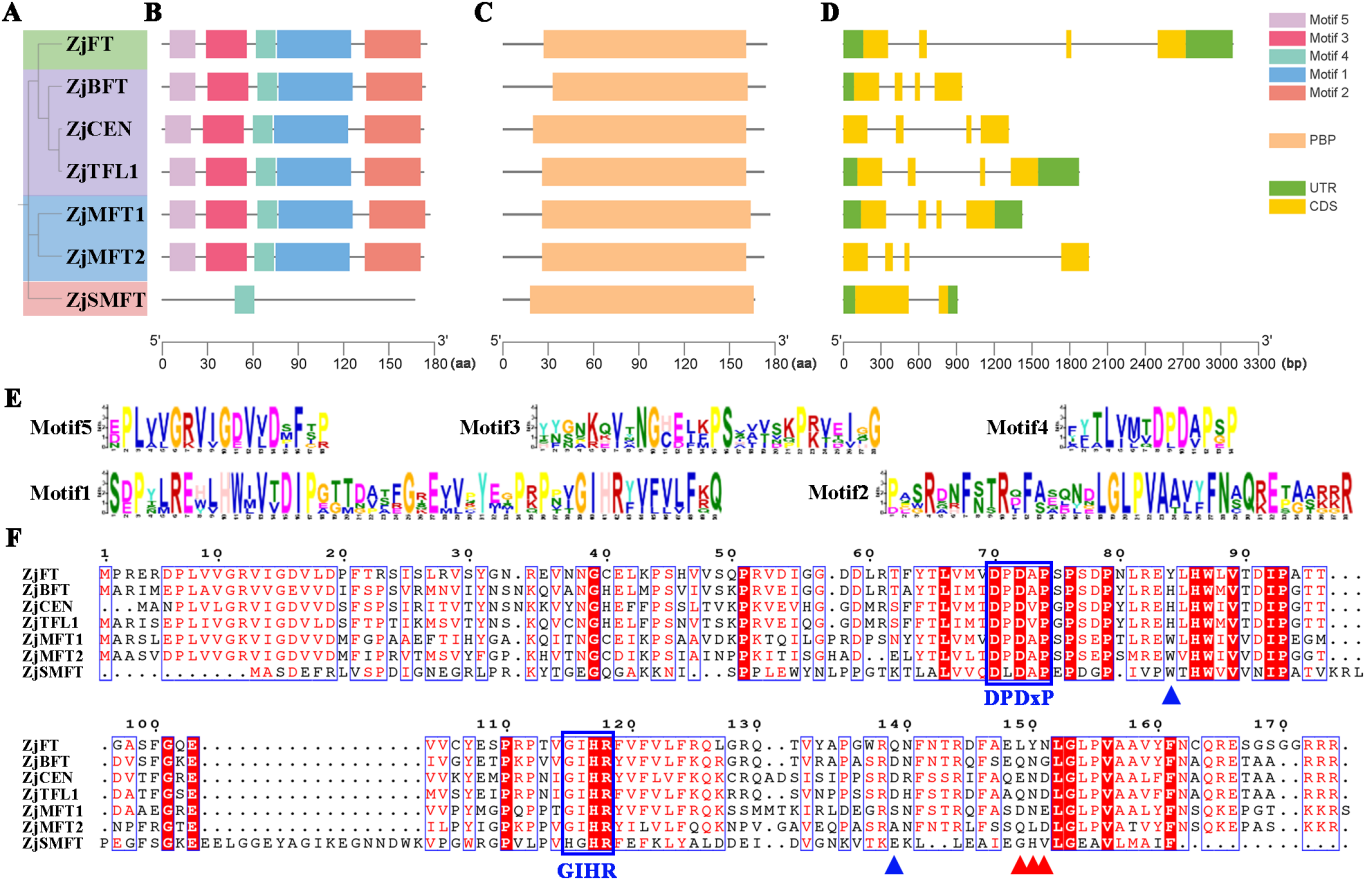
Phylogenetic relationships, gene structural features, conserved motifs, and protein sequence alignment of ZjPEBPs. **(A)** Phylogenetic tree of ZjPEBP gene family members. **(B)** Conserved motifs in ZjPEBP proteins. Color-coded boxes represent motifs 1-5. **(C)** Conserved domains in ZjPEBP proteins. **(D)** Exon-intron structures of ZjPEBP genes. Green: UTRs; Yellow: CDS; Black lines: introns. **(E)** Sequence logos of conserved motifs in ZjPEBP proteins. **(F)** Amino acid sequence alignment of ZjPEBP family members. Blue boxes: DPDxP and GIHR motifs; Blue triangles: key residues determining FT/TFL1 functional specificity; Red triangles: LYN triad.

A phylogenetic tree comprising exclusively sour jujube ZjPEBP members was constructed using MEGA 11.0 (**Figure 2A**), showing topology consistent with the multi-species phylogeny (**Figure 1**). Five conserved motifs were identified in ZjPEBP proteins (**Figures 2B**, 2E). All members except ZjSMFT contained motifs 1–5 with identical sequential arrangement, while ZjSMFT contained only motif 4. Analysis of conserved domains confirmed the presence of the characteristic PEBP domain in all ZjPEBP family members (**Figure 2C**). Gene structure analysis revealed that ZjSMFT exhibits a two-exon/one-intron organization, whereas other members uniformly display a four-exon/three-intron structure (**Figure 2D**). Structural variations primarily occurred in the lengths of 5’ and 3’ untranslated regions (UTRs) and intronic sequences.

Multiple protein sequence alignment demonstrated high conservation of key functional motifs: DPDxP (Asp-Pro-Asp-X-Pro) and GIHR (Gly-Ile-His-Arg) across ZjPEBP members (**Figure 2F**). Crucially, our sequence alignment showed that key amino acid residues governing functional divergence between FT and TFL1 clades—specifically His^88^/Tyr^69^ and Asp^76^/Gln¹^40^—were conserved in sour jujube. Furthermore, the LYN triad (Leu-Tyr-Asn), an established diagnostic feature for FT functionality (Hanzawa et al., 2005; Ahn et al., 2006), was also strictly conserved in the ZjFT. These findings indicate evolutionary conservation of molecular mechanisms underlying PEBP-mediated flowering regulation in sour jujube.

### 3.4 Gene duplication events and collinearity analysis of *ZjPEBP* family

Gene duplication represents a primary driver of gene family expansion and functional diversification. To identify duplication modes underlying *ZjPEBP* family expansion, genome-wide analysis of duplication types was performed, including singleton, dispersed, proximal, tandem and WGD/segmental. Results demonstrated that *ZjSMFT* originated from singleton duplication, while the remaining six *ZjPEBP* genes derived from dispersed duplication events. This indicates dispersed duplications served as the predominant mechanism for *ZjPEBP* family expansion in sour jujube.

**Figure 3:**
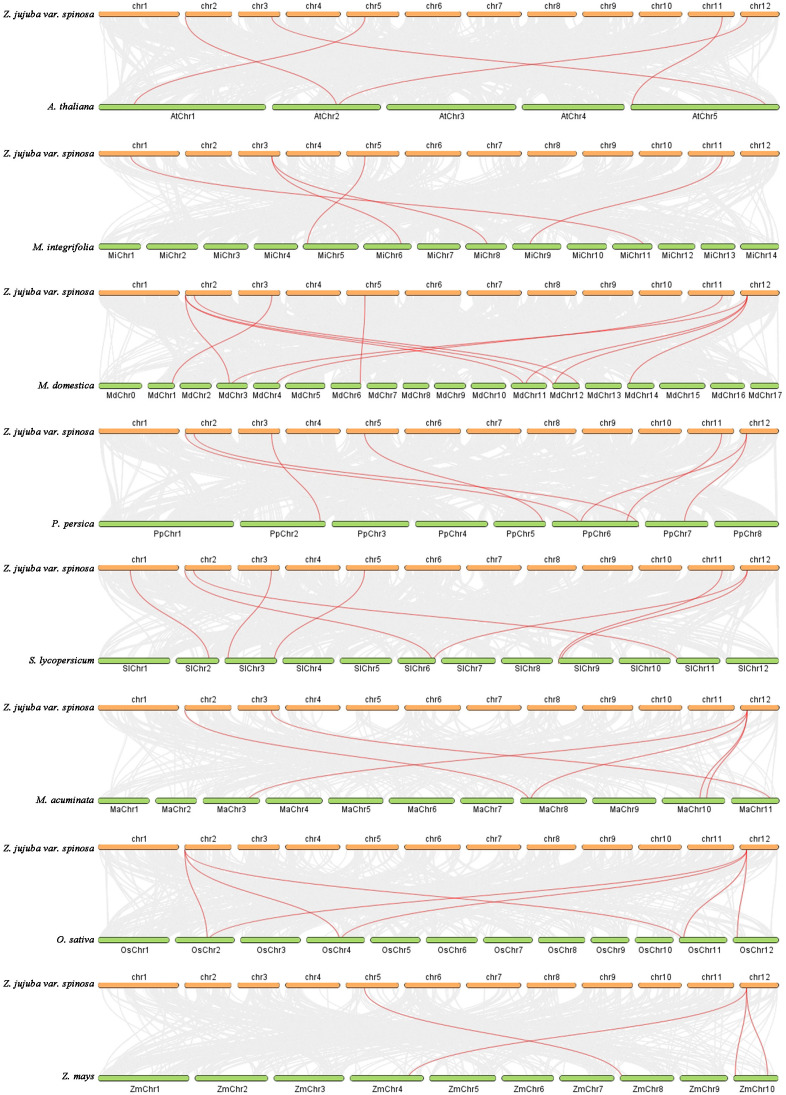
Collinearity analysis of *PEBP* genes between sour jujube and other plant species. Gray lines indicate collinear genomic regions; Red lines highlight syntenic *PEBP* homologous gene pairs.

Collinearity analysis revealed no intra-species collinear relationships among *ZjPEBP* members. To further investigate evolutionary mechanisms, inter-species collinearity analysis was conducted between sour jujube and other plant species, utilizing the same angiosperm species employed in our phylogenetic reconstruction. Specifically, the numbers of homologous *PEBP* gene pairs between sour jujube and the five dicot species (*A. thaliana, M. integrifolia, M. domestica, P. persica, S. lycopersicum*) were 5, 5, 11, 7, and 8, respectively. The numbers of homologous gene pairs with the three monocot species (*M. acuminata, O. sativa, Z. mays*) were 6, 7, and 4, respectively (**Figure 3**, **Supplementary Table S5**). Notably, sour jujube exhibited the highest collinearity with apple, further supporting close phylogenetic relationships between *Ziziphus* and Rosaceae species.

### 3.5 Cis-element analysis of *ZjPEBP* promoters

**Figure 4:**
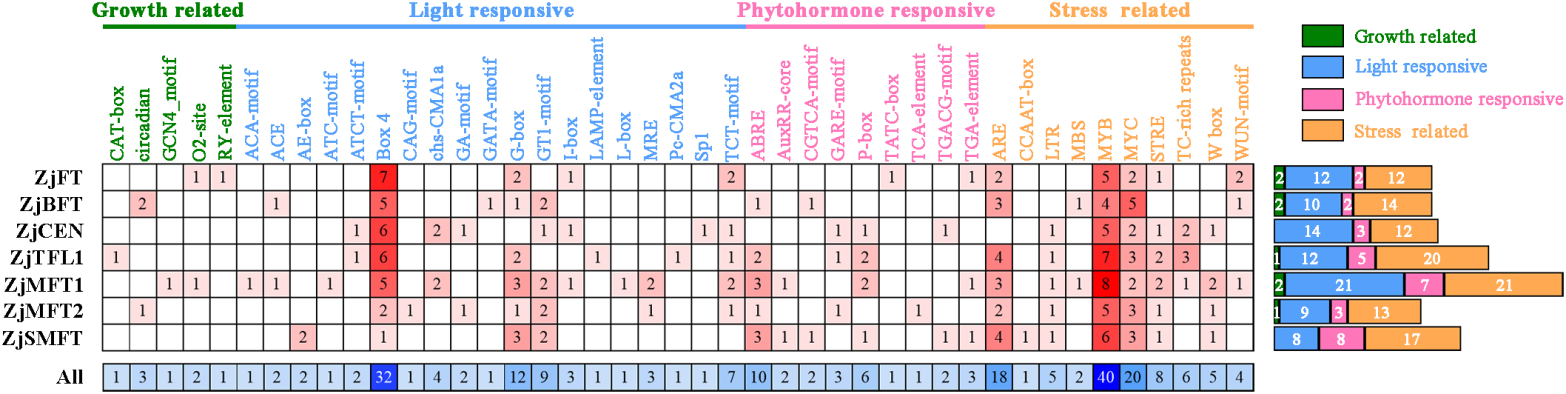
Cis-element analysis in promoter regions of *ZjPEBP*. Color-coded blocks represent four functional categories of cis-elements. Horizontal lines demarcate individual genes. Numerical annotations indicate element counts per gene.

Cis-regulatory elements play critical roles in transcriptional regulation. To investigate the potential molecular mechanisms underlying flowering regulation by *ZjPEBP* genes in sour jujube, promoter sequences spanning 2000 bp upstream of start codons were analyzed for cis-element composition (**Supplementary Table S2**). A total of 43 cis-regulatory elements were identified in sour jujube *ZjPEBP* promoters, categorically classified into four functional groups: growth and development-associated elements, light-responsive elements, hormone-responsive elements, and stress-responsive elements (**Figure 4**, **Supplementary Table S6**). Light-responsive elements constituted the most abundant category and were present in all promoters, highlighting the pivotal role of light signaling in regulating PEBP gene function. Among stress-responsive elements, transcription factor binding sites (MYB and MYC) occurred universally, suggesting these transcriptional regulators may control *ZjPEBP* expression. Most promoters additionally contained antioxidant response elements (ARE) and stress-responsive elements (STRE). In hormone-responsive elements, abscisic acid response elements (ABRE) and gibberellin-responsive elements (P-box) were more prevalent than other phytohormone-related motifs. The ubiquitous occurrence of stress- and hormone-responsive elements implies that the *ZjPEBP* gene family may participate in diverse stress adaptation and phytohormone signaling pathways.

### 3.6 Tissue-specific and spatiotemporal expression patterns of *ZjPEBP* genes

**Figure 5:**
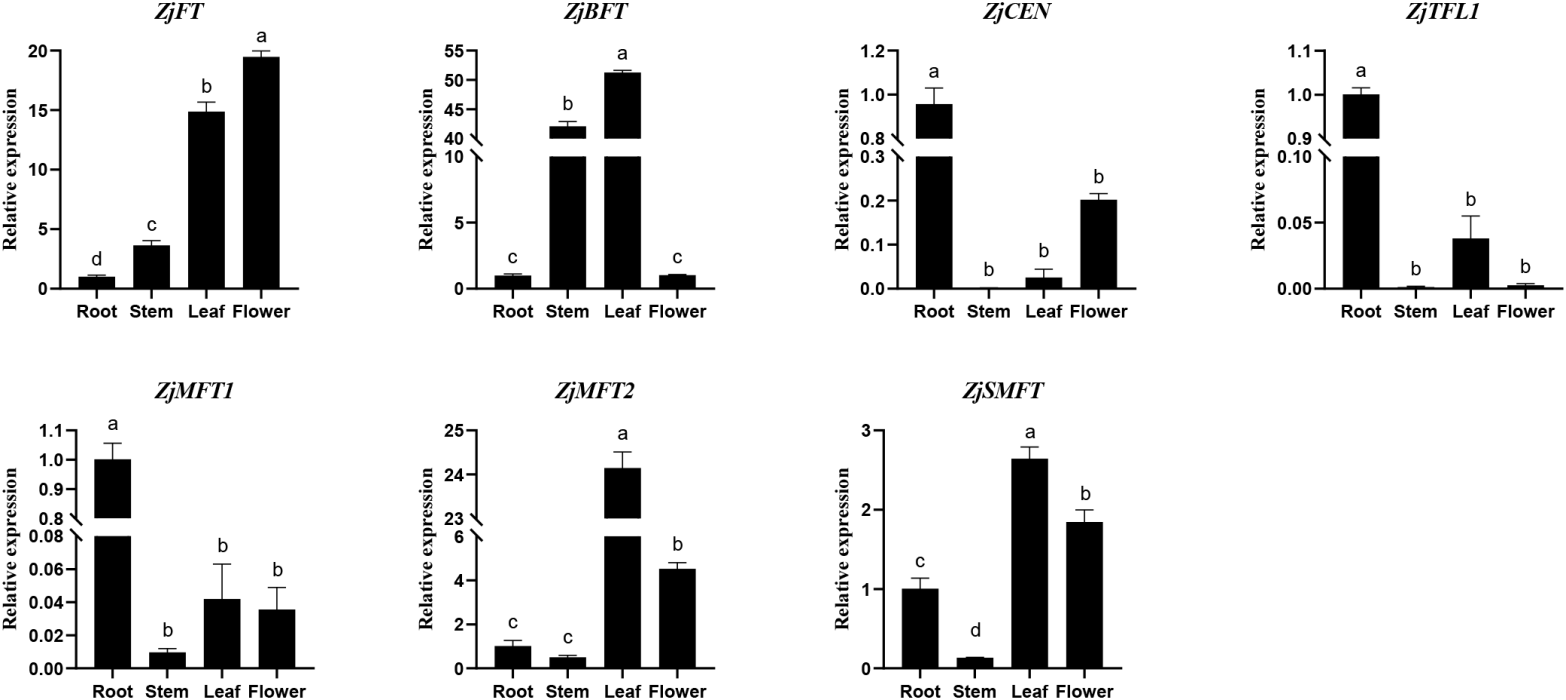
Tissue-specificexpression of *ZjPEBP* genes. Tissue-specific expression patterns of *ZjPEBP* genes. Gene expression levels are presented relative to the expression level in root tissues for each respective gene. All RT-qPCR data were normalized to the Actin reference gene and analyzed using the 2^–ΔΔCt^ method. Data represent mean ± SD of three biological replicates. Statistical differences are indicated by different lowercase letters (P < 0.05) according to one-way ANOVA followed by Tukey’s HSD test.

To detect expression differences among *ZjPEBP* gene family, their expression patterns were analyzed across in root, stem, leaf, and floral tissues using RT-qPCR (**Figure 5**). Distinct tissue-specific expression was observed: *ZjFT*, *ZjMFT2* and *ZjSMFT* predominated in leaves and flowers; *ZjCEN*, *ZjTFL1* and *ZjMFT1* primarily expressed in roots; while *ZjBFT* showed dominant expression in stems and leaves. This member-specific expression partitioning suggests functional diversification within the *ZjPEBP* family during sour jujube evolution.

**Figure 6:**
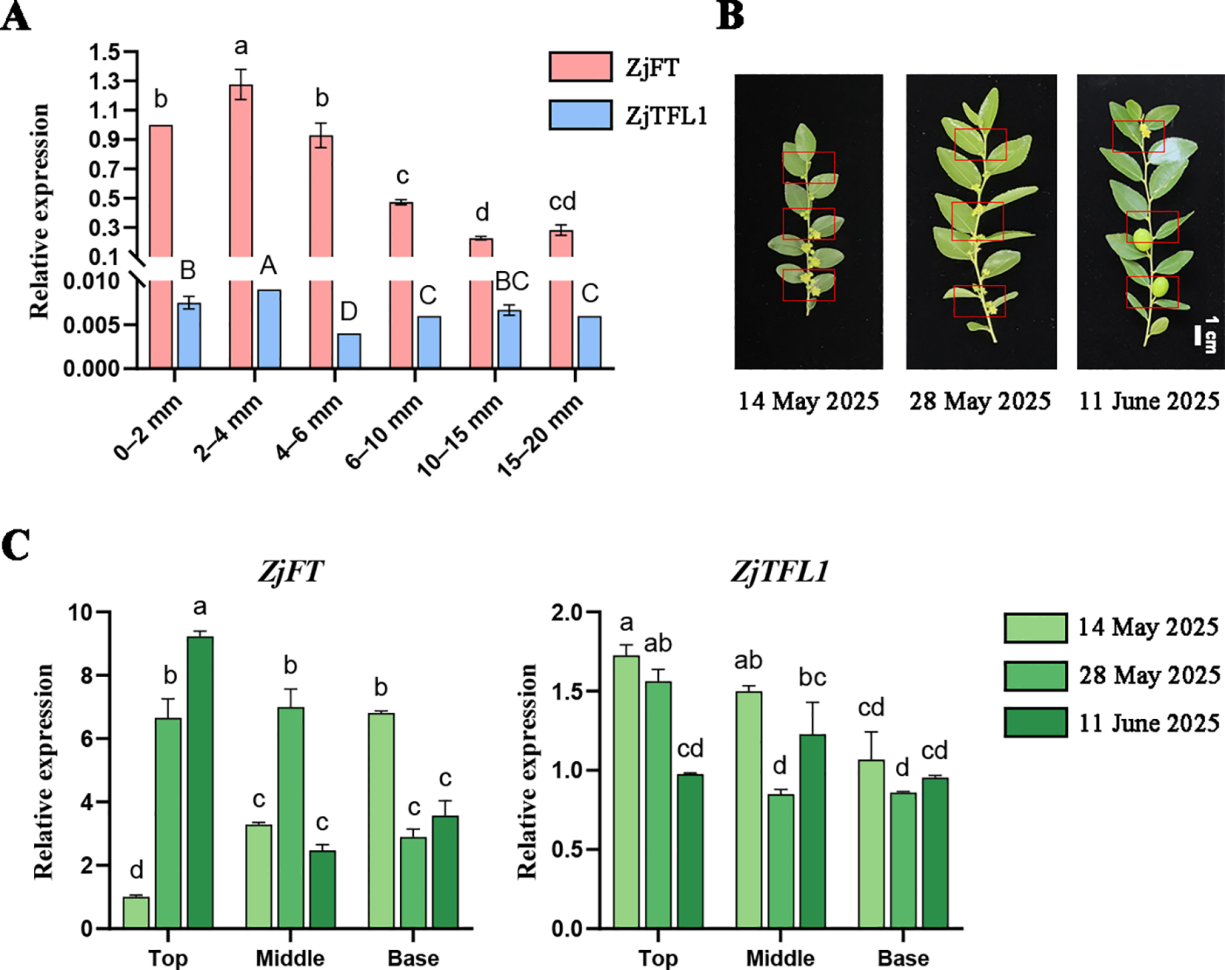
Spatiotemporal expression patterns of *ZjFT* and *ZjTFL1* during FBS development. **(A)** Expression kinetics of *ZjFT* and *ZjTFL1* during FBS elongation. Expression levels are calculated relative to the expression level of *ZjFT* in 0–2 mm FBS. **(B)** FBS at differential developmental stages with sampling sites. Red boxes highlight Top, Middle, and Base sampling positions sequentially from top to bottom. **(C)** Temporospatial expression patterns of *ZjFT* and *ZjTFL1* in leaves from different FBS sections. Expression levels are shown relative to the expression level of *ZjFT* in the top section of FBS at the initial time point. Gradient green represents distinct developmental stages of FBS; Top, Middle, and Base denote leaves from apical, middle, and basal FBS sections, respectively. All RT-qPCR data were normalized to the Actin reference gene and analyzed using the 2^–ΔΔCt^ method. Data represent mean ± SD of three biological replicates. Statistical differences are indicated by different lowercase letters (P < 0.05) according to one-way ANOVA followed by Tukey’s HSD test.

Expression levels of *FT*/*TFL1* play pivotal roles in flowering time determination, with floral bud differentiation in sour jujube initiating at 2–3 mm FBS length. Therefore, to decipher spatiotemporal expression patterns of *ZjFT*/*ZjTFL1* during FBS development, we conducted RT-qPCR analysis across differentially elongated FBS (**Figure 6A**). Both genes exhibited peak expression at 2–4 mm FBS followed by progressive decline, though *ZjTFL1* maintained significantly lower expression than *ZjFT* at all developmental stages. During subsequent elongation of FBS, floral differentiation progressed basipetally from the base toward the top until elongation ceased. Consequently, spatiotemporal expression profiling was performed on leaves from apical, middle, and basal FBS sections post-flowering (**Figure 6B, C**). During floral anthesis, spatial analysis revealed elevated *ZjFT* expression in base sections relative to middle and top sections, while *ZjTFL1* exhibited an inverse expression gradient, with its highest expression in the top sections. Upon fruit set in base sections, both genes were immediately downregulated. When examining temporal dynamics within each section, the top sections undergoing progressive flower opening exhibited significant *ZjFT* upregulation concomitant with *ZjTFL1* downregulation. Middle sections transitioning from flowering to fruit formation displayed opposite expression patterns: *ZjFT* initially increased then decreased, while *ZjTFL1* reciprocally decreased then increased. Throughout the developmental progression of base sections from flowering through fruit set and expansion, a sustained downregulation of *ZjFT* was observed, whereas *ZjTFL1* expression showed no detectable changes.

### 3.7 ABA-responsive expression patterns of *ZjPEBP* genes

**Figure 7:**
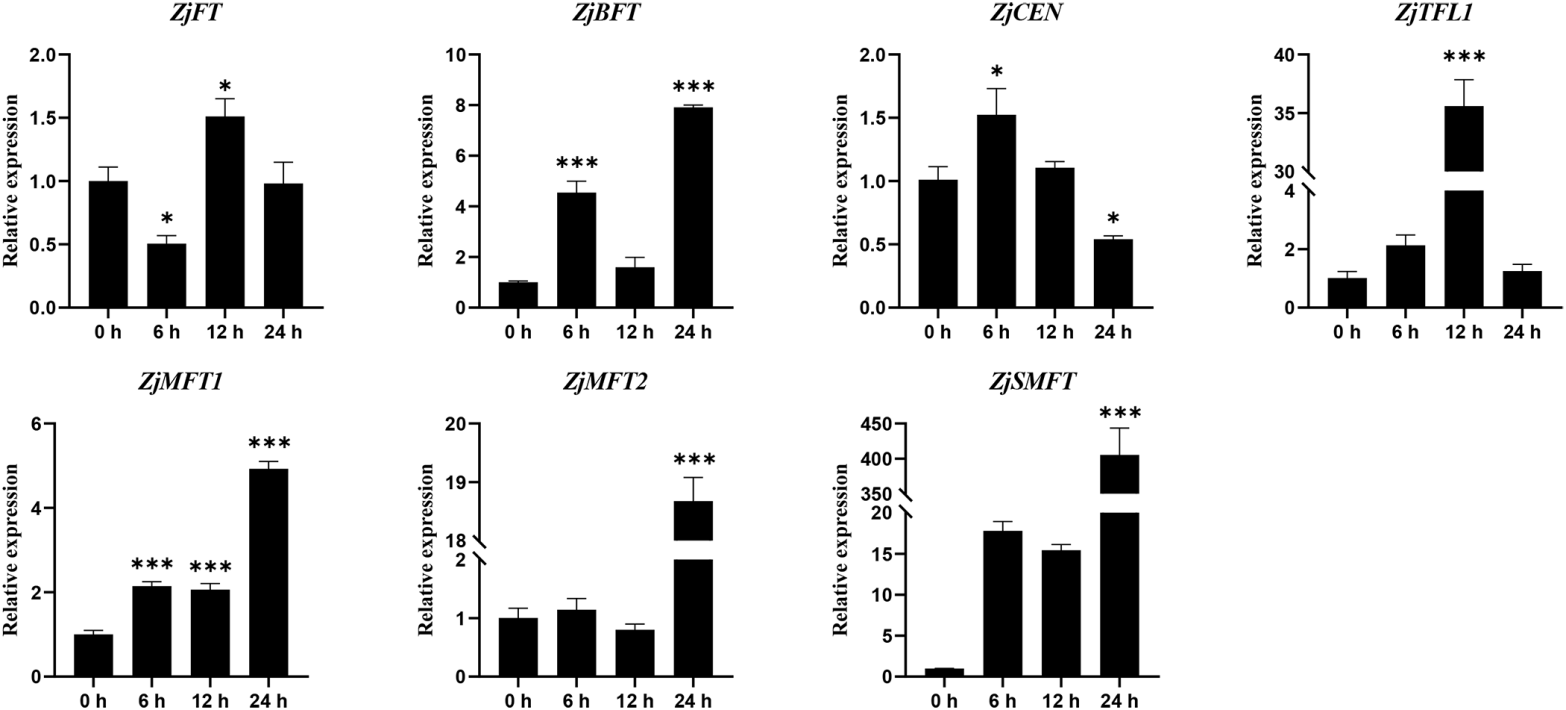
Temporal expression profiles of *ZjPEBP* genes under 100 μM ABA treatment. Gene expression levels normalized to the 0 h time point for each gene. Data represent mean ± SD of three biological replicates. Statistical differences are indicated by asterisks (*P < 0.05, ***P < 0.001) according to one-way ANOVA followed by Tukey’s HSD test.

As a key phytohormone regulating development and stress adaptation, ABA potentially modulates *ZjPEBP* expression given the prevalence of ABRE in their promoters. RT-qPCR analysis of leaves from sour jujube seedlings treated with 100 μM ABA revealed significant temporal dynamics and amplitude variations in the response of *ZjPEBP* family genes to ABA (**Figure 7**). Specifically, the expression of *ZjBFT*, *ZjMFT1*, *ZjMFT2* and *ZjSMFT* was strongly induced, showing sustained upregulation and reaching their highest observed expression levels at the 24 h time point within the experimental period. Notably, *ZjSMFT* expression was sharply upregulated by approximately 400-fold, demonstrating extremely high sensitivity to ABA. In contrast, the expression of *ZjFT* was transiently suppressed at 6 h, gradually recovered thereafter, and returned to near baseline levels by 24 h. *ZjCEN* showed transient upregulation at 6 h before significant downregulation at 12 h, ultimately falling below its 0 h level by 24 h, while *ZjTFL1* expression peaked at 12 h before declining. These results demonstrate that *ZjPEBP* family members are responsive to ABA signaling and undergo significant transcriptional reprogramming upon ABA exposure.

## 4 Discussion

Flowering is a critical developmental transition from vegetative to reproductive growth, with PEBP gene family members playing pivotal roles in regulating flowering time and plant architecture (Corbesier et al., 2007; Jung and Müller, 2009; Amasino, 2010). Gene duplication events during evolution result in interspecies variation in *PEBP* gene numbers. Typically, dicot species harbor fewer *PEBP* genes than monocots. For instance, the dicot models *A. thaliana* and *M. domestica* contain 7 and 8 *PEBP* genes respectively (Wickland and Hanzawa, 2015; Zhang et al., 2021; Bellinazzo et al., 2024), whereas monocots *O. sativa* and *Z. mays* possess 20 and 25 genes (Danilevskaya et al., 2008; Zhao et al., 2022). In this study, we identified seven *ZjPEBP* genes in the sour jujube genome, classified into four subfamilies: *MFT*-like (*ZjMFT1*, *ZjMFT2*), *FT*-like (*ZjFT*), *TFL1*-like (*ZjCEN*, *ZjBFT*, *ZjTFL1*), and *SMFT*-like (*ZjSMFT*) (**Figure 1**, **Table 1**). This phylogenetic classification is consistent with the organizations of the PEBP family reported in other species (He et al., 2022; Fan et al., 2024). Furthermore, both phylogenetic and collinearity analyses revealed closer homology between sour jujube ZjPEBP and Rosaceae species (**Figures 1**, **3**), supporting the established closer evolutionary relationship between Rhamnaceae and Rosaceae documented in genomic evolutionary studies (Liu et al., 2014). Notably, our phylogenomic analysis placed ZjSMFT within a prokaryote-dominant clade (**Figure 1**) with significant sequence conservation to bacterial YbhB proteins (**Supplementary Figure S4**). Gene duplication analysis indicated that *ZjSMFT* originated from singleton duplication, supporting the hypothesis of its origin via HGT from prokaryotes (Bellinazzo et al., 2024). The remaining six *ZjPEBP* genes derived from dispersed duplication events, indicating that dispersed duplications served as the primary driver of *ZjPEBP* family expansion and functional diversification in sour jujube.

Structural analysis of *ZjPEBP* genes revealed conserved organizational features. Classical *PEBP* family members typically exhibit a conserved four-exon gene structure (Danilevskaya et al., 2008). In sour jujube, six *ZjPEBP* genes maintained the characteristic four-exon/three-intron organization, whereas *ZjSMFT* displayed a divergent two-exon/one-intron structure (**Figure 2D**). At the protein level, this six conserved ZjPEBPs contained five identically ordered motifs, including the preserved DPDxP and GIHR functional domains, confirming their evolutionary conservation (**Figure 2B, F**). In contrast, ZjSMFT retained only motif 4 (**Figure 2B**). Despite divergence in gene structure and motif composition, *ZjSMFT* encodes a complete PEBP domain (**Figure 2C**), and its structural distinctions may be associated with HGT events (Tsoy and Mushegian, 2022; Bellinazzo et al., 2024). The molecular functions of *PEBP* genes are well-established across plant species, where *FT* and *TFL1* serve as antagonistic molecular switches regulating flowering time (Kobayashi et al., 1999). In *A. thaliana*, functional reversal between FT and TFL1 occurs through single-amino acid substitutions at critical positions (Tyr^85^/His^88^ and Gln¹^40^/Asp¹^44^) (Hanzawa et al., 2005; Ahn et al., 2006). Protein sequence alignment demonstrated strict conservation of these functional determinant residues in sour jujube homologs: Tyr^80^/His^80^ and Gln¹³^5^/Asp¹³^5^ in ZjFT/ZjTFL1 respectively (**Figure 2F**). Additionally, the diagnostic LYN triad essential for FT functionality was conserved in ZjFT (Ahn et al., 2006).

Tissue-specific gene expression patterns often reflect functional differentiation. Our expression profiling of seven *ZjPEBP* genes revealed pronounced organ specificity (**Figure 5**): *ZjFT*, *ZjMFT2*, and *ZjSMFT* showed preferential expression in leaves and flowers; *ZjMFT1*, *ZjCEN*, and *ZjTFL1* accumulated predominantly in roots; while *ZjBFT* exhibited high expression in stems and leaves. This differential expression pattern demonstrates functional divergence among *ZjPEBP* family members. To elucidate the molecular regulatory mechanism underlying the “continuous differentiation” flowering pattern in sour jujube, this study systematically analyzed the expression dynamics of key flowering regulators *ZjFT* and *ZjTFL1* during the development of FBS (**Figure 6**). In the early stage of FBS development (**Figure 6A**), both *ZjFT* and *ZjTFL1* reached their expression peaks when FBS elongated to 2–4 mm, a period that aligns precisely with the initial stage of floral bud morphological differentiation (Qu and Wang, 1993). Notably, although their expression trends were similar, the expression level of *ZjTFL1* was significantly lower than that of *ZjFT* at all detected stages, resulting in a pronounced expression imbalance. This molecular pattern—dominant expression of *ZjFT* coupled with relative suppression of *ZjTFL1*—provides a crucial initial regulatory signal for the continuous floral bud differentiation in sour jujube. This finding is consistent with the research results reported by Cardon et al. in coffee, whose study demonstrated that the expression window of *CaFT1* overlaps with the entire process of floral bud development (Cardon et al., 2022), further confirming the core regulatory role of *FT* homologs in mediating asynchronous flowering behavior in perennial plants. As FBS elongation continued, floral bud differentiation progressed progressively from the base to the apex (**Figure 6B**). At the first sampling time point, *ZjFT* expression in basal leaves was significantly higher than in the middle and upper regions, whereas *ZjTFL1* exhibited an inverse expression gradient. This spatial distribution pattern corresponded closely with the process of floral bud differentiation and resembled the *FcFT* expression pattern previously reported in fig (Ikegami et al., 2013). In the apical region of FBS, where floral bud differentiation is ongoing (**Figure 6C**), *ZjFT* expression gradually increased while *ZjTFL1* was concurrently downregulated as leaves matured and floral organs developed, thereby sustaining the continuous differentiation of the inflorescence meristem. Conversely, in the middle and basal regions that had entered the fruit-setting stage, inflorescence differentiation tends to cease with advancing fruit development (Ware et al., 2020). Correspondingly, *ZjFT* in these areas shows rapid downregulation, marking the termination of floral organ differentiation. However, this continuous differentiation strategy imposes significant physiological burdens on sour jujube. The persistent formation of floral organs consumes substantial storage nutrients, which represents a major cause of severe flower and fruit drop in jujube trees, resulting in a fruit-setting rate below 1%. In-depth analysis of *ZjFT* expression patterns in sour jujube and precise regulation of its spatiotemporal dynamics will contribute to optimizing the flowering mechanism, thereby establishing a foundation for improving fruit-setting rate through molecular breeding approaches.

Analysis of *ZjPEBP* promoter architectures revealed abundant cis-regulatory elements spanning four functional categories: growth-related, light responsiveness, hormone signaling, and stress responses (**Figure 4**). Divergent element composition and density among homologs suggest functional diversification across signaling pathways. Notably, several promoters contained ABA-responsive elements (ABRE, MYB and MYC), which are known to mediate ABA-dependent abiotic stress responses (Busk and Pagès, 1998; Yamaguchi-Shinozaki and Shinozaki, 2005). Such elements are commonly present in *PEBP* family genes across multiple species. For instance, promoters of rice *OsPEBP* family members also harbor multiple ABA-responsive elements, and their expression is induced by ABA (Zhao et al., 2022). Additionally, ABRE elements are found in the promoters of *MFT* genes in species such as *A. thaliana*, rice, and cotton, where *MFT* acts as an antagonist of ABA signaling during seed germination (Xi et al., 2010; Yu et al., 2019). These findings suggest that the involvement of *PEBP* genes in ABA-mediated physiological processes through promoter ABREs may represent a relatively conserved regulatory mechanism in plants. As a key hormone in plant responses to abiotic stress, ABA not only mediates adaptation to drought, osmotic, and salt stress but also plays a complex role in flowering time regulation, exhibiting both promotive and inhibitory effects (Koops et al., 2011; Wang et al., 2013; Riboni et al., 2016; Robustelli Test et al., 2025). For example, in *A. thaliana*, salt stress upregulates *BFT* expression via an ABA-dependent pathway, leading to delayed flowering under high salinity (Chung et al., 2010; Ryu et al., 2011). Similarly, the rice *RCN1* gene is induced by drought stress in an ABA-dependent manner and delays heading under drought conditions (Wang et al., 2020). Although ABA treatment generally suppresses flowering, Koops et al. found that root-applied ABA promotes flowering in *A. thaliana*. This treatment mimics the positive role of endogenous ABA in flowering by affecting the signaling of photoperiod-related genes such as *GIGANTEA (GI)* and *SUPPRESSOR OF OVEREXPRESSION OF CONSTANS1 (SOC1)*, thereby activating the key flowering gene *FT* (Riboni et al., 2013; Riboni et al., 2016). To validate the functional relevance of ABA-responsive elements in *ZjPEBP* promoters, we analyzed the expression dynamics of this gene family under ABA treatment using RT-qPCR (**Figure 7**). The results showed that all *ZjPEBP* members responded to ABA treatment but exhibited distinct temporal expression patterns. Among them, *ZjSMFT* exhibited exceptionally strong upregulation, reports that its orthologs are involved in biotic and abiotic stress responses (Burton et al., 2025). These findings suggest that the *ZjPEBP* gene family possesses transcriptional regulatory mechanisms for responding to ABA signals, and its members may potentially function as regulators in ABA signaling pathways. However, further functional studies are needed to confirm their precise role in ABA-mediated stress adaptation in sour jujube.

## 5 Conclusion

In this study, we identified seven *ZjPEBP* genes in the *Z. jujuba* var. *spinosa* genome. Phylogenetic analysis classified them into four subfamilies: *TFL1*-like, *FT*-like, *MFT*-like, and *SMFT*-like. The *SMFT*-like member *ZjSMFT* likely originated via horizontal gene transfer from prokaryotes, exhibited high sequence homology with bacterial genes. The remaining members expanded via dispersed duplication events while maintaining conserved gene structures and protein motifs. Cis-element analysis revealed abundant light-responsive and ABA-responsive elements in *ZjPEBP* promoters. RT-qPCR analysis revealed tissue-specific expression patterns of *ZjPEBP* genes and their differential responses to ABA treatment. Crucially, the expression dynamics of *ZjFT* correlated with floral bud differentiation progression in FBS. These findings establish a foundation for deciphering the molecular mechanisms whereby *ZjPEBP* genes regulate flowering in sour jujube.

## Data Availability

The original contributions presented in the study are included in the article/**Supplementary Material**. Further inquiries can be directed to the corresponding author.
