# Supplementary material for: Genome-wide identification and expression analysis of the PEBP gene family in Ziziphus jujuba var. spinosa
Source: Front Plant Sci. 2026 Jan 5;16:1700555. doi: 10.3389/fpls.2025.1700555 (PMC12812895; doi:10.3389/fpls.2025.1700555)
Supplement: Supplementary file 1 [file DataSheet1.docx]

Supplementary Material


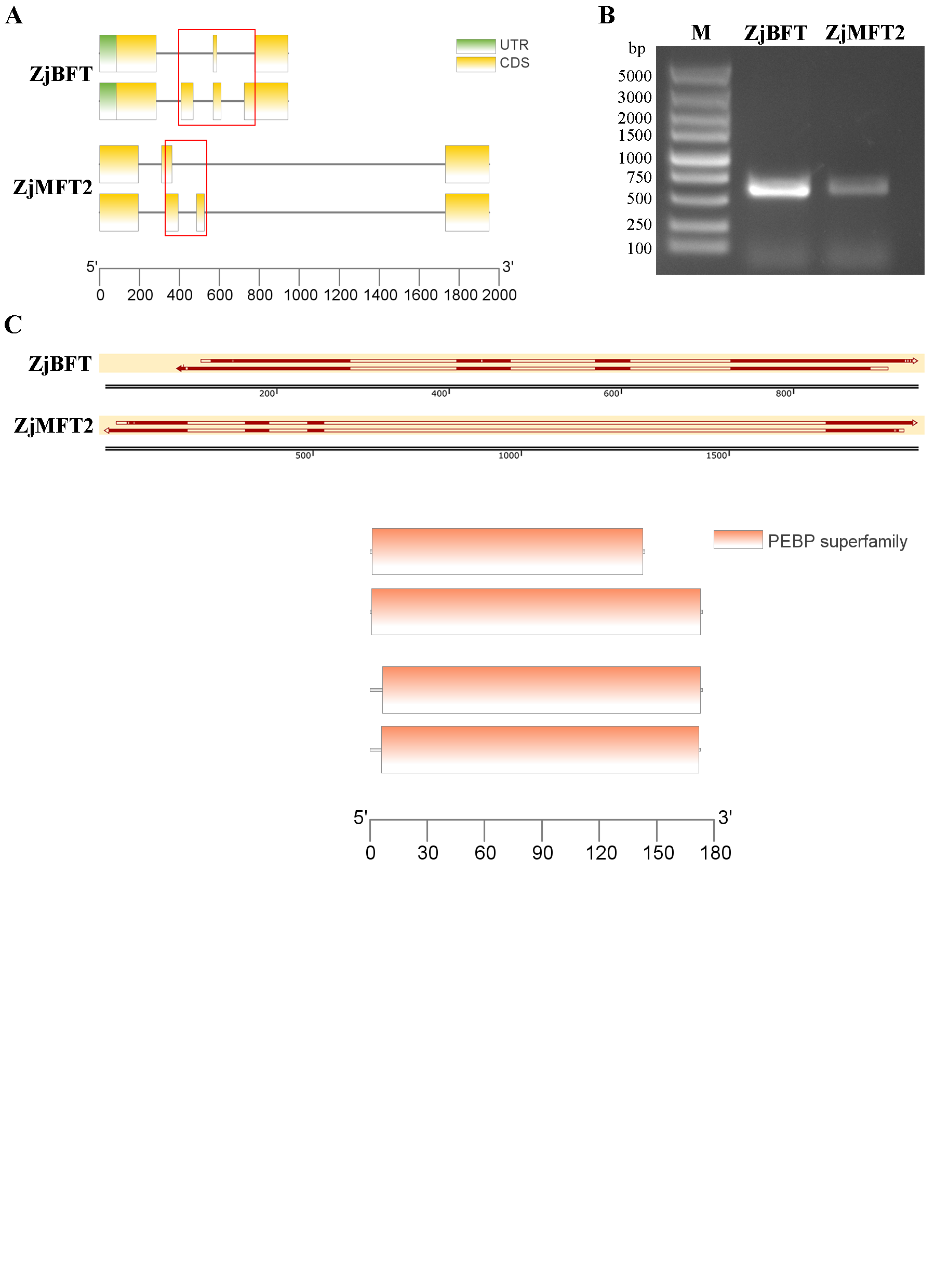


**Supplementary Figure 1.** **Validation and structural correction of *ZjBFT* and *ZjMFT2* sequences.** (A) Gene structure schematics showing the corrected regions (highlighted by red rectangles), as validated by PCR and sequencing. (B) Electropherogram of PCR products amplified from sour jujube cDNA. M: DNA Marker. (C) Sequence alignment of PCR-amplified fragments against the genomic reference, confirming the corrected coding sequences.


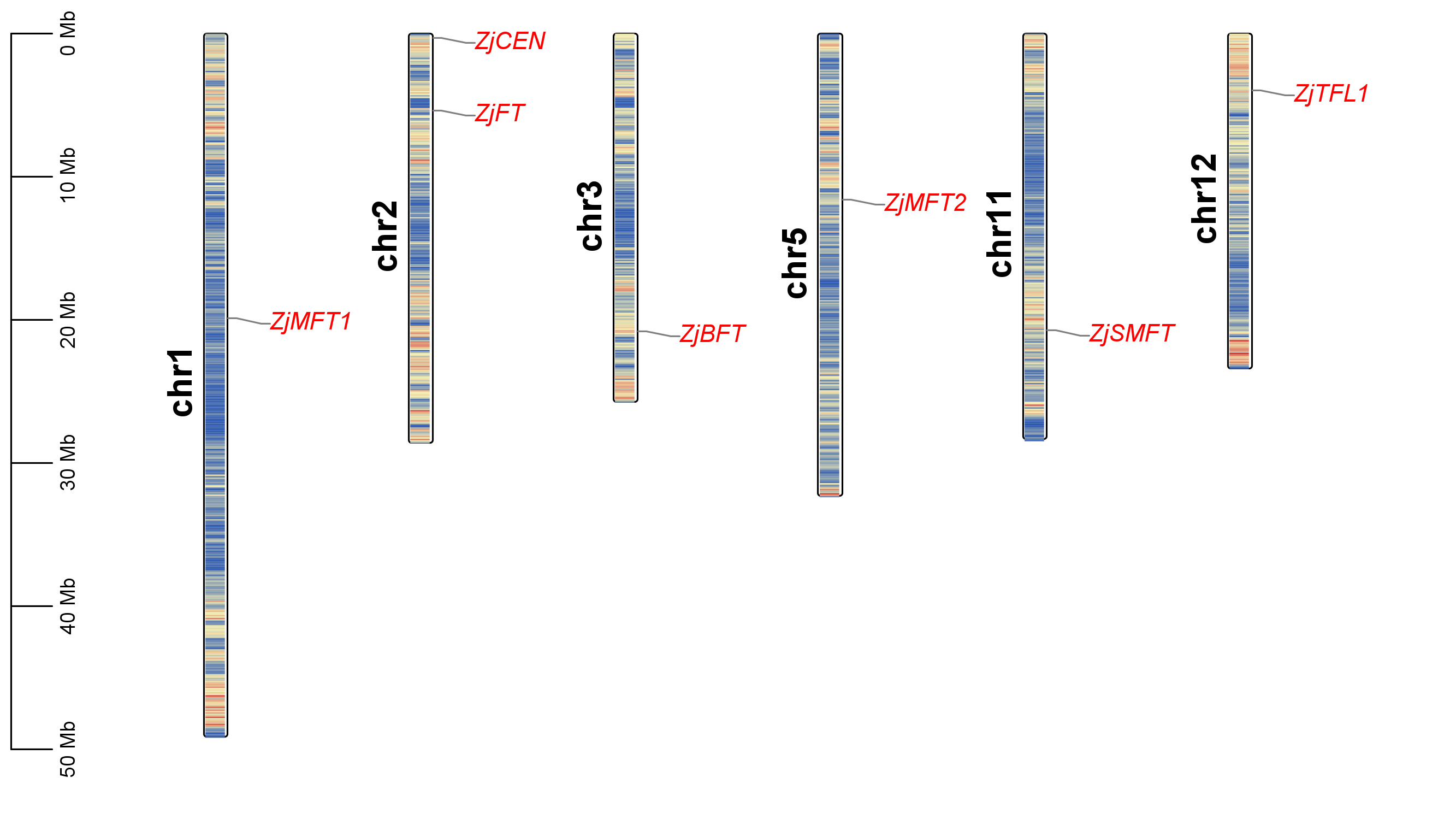


**Supplementary Figure 2.** **Chromosomal localization of the *ZjPEBP* in *Z. jujuba* var. *spinosa.*** The scale bar on the left estimates chromosome length. The elongated bars represent *Z. jujuba* var. *spinosa* chromosomes. Numbers on the left side of the chromosomes indicate chromosome numbers. The gradient from blue to red on the chromosomes represents gene density. The *ZjPEBP* gene is highlighted in red on the right side of the chromosome.


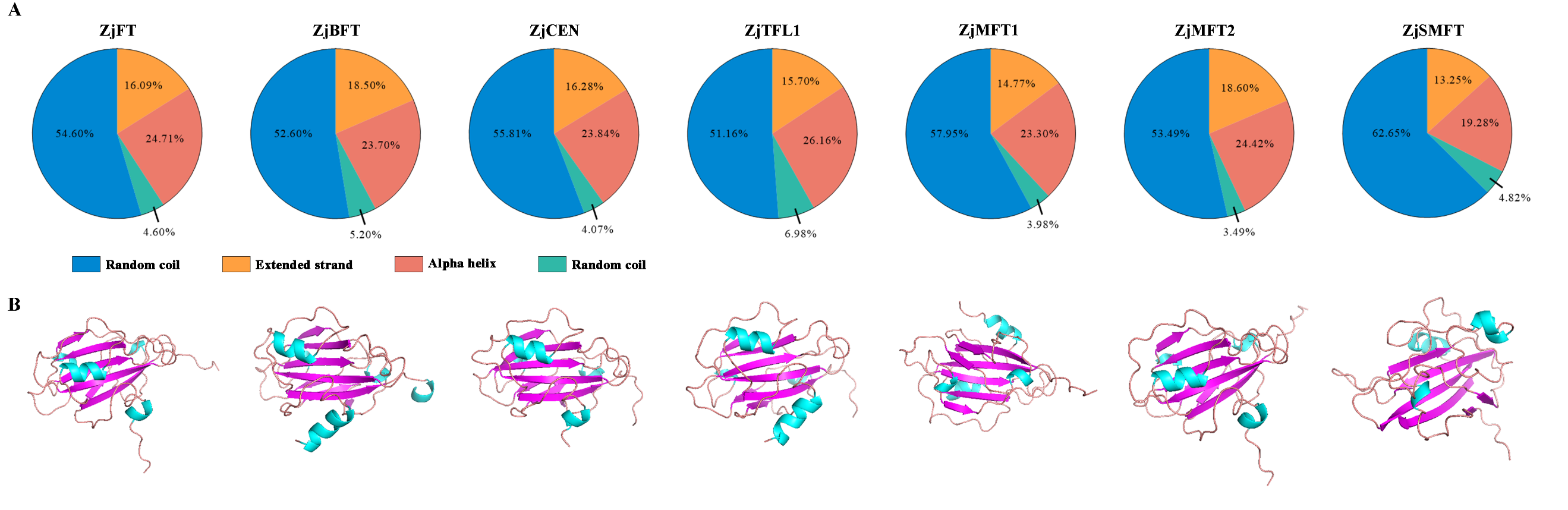


**Supplementary Figure 3.** **Secondary and tertiary structures of the ZjPEBP protein from *Z. jujuba* var. *spinosa.*** (A) Secondary structure of ZjPEBP protein. (B) Tertiary structure of ZjPEBP protein.


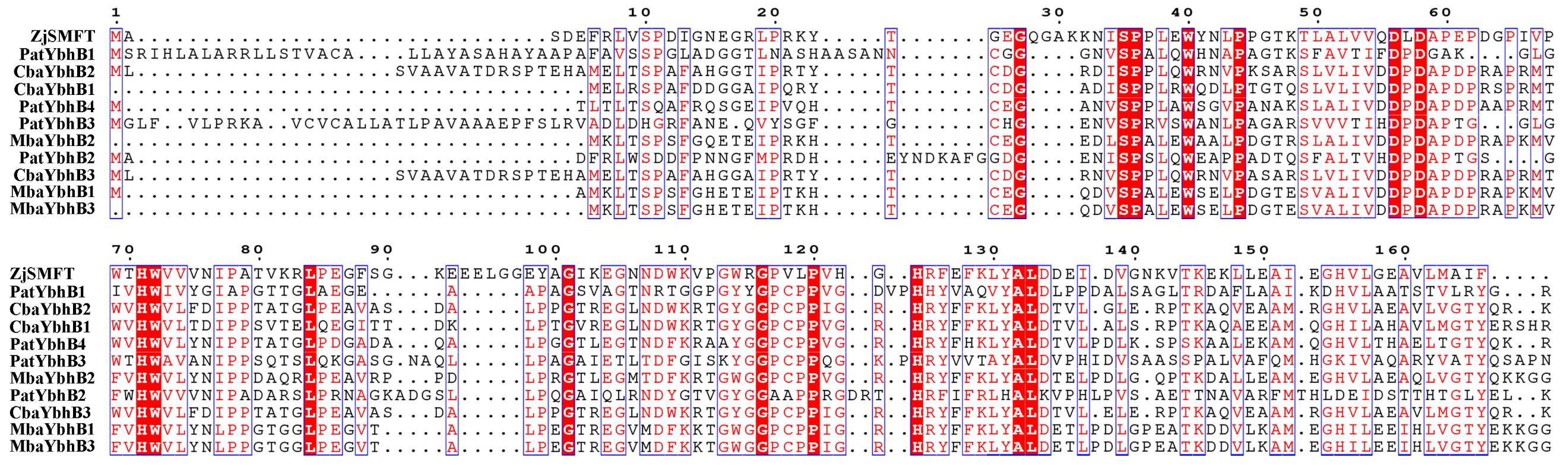


**Supplementary Figure 4.** **Protein sequence alignment of ZjSMFT and bacterial YbhB.**
